# Linking Women’s Empowerment to Mental Health Symptoms: Population-Based Study

**DOI:** 10.2196/85839

**Published:** 2026-09-08

**Authors:** Maggie Craig, Hannah Walter, Masum Ali, Shahriar Faruque, Sanjib Saha

**Affiliations:** 1 Department of Clinical Sciences (Malmö), Lund University, BMC, Sölvegatan 19, Lund, 22362, Sweden, 46 724674880; 2 Nutrition and Health Science, Laney Graduate School, Emory University, Atlanta, GA, United States; 3 Adult Psychiatry, Directorate General of Health, Directorate General of Health, Dhaka, Bangladesh

**Keywords:** women’s empowerment, mental health, Bangladesh, depression, anxiety, Survey-Based Women’s Empowerment Global index, SWPER Global index, intimate partner violence, IPV, low- and middle-income countries, LMICs, demographic and health survey

## Abstract

**Background:**

Mental health disorders represent a growing global challenge, particularly in low- and middle-income countries, where women are disproportionately affected by social and structural inequalities. Although women’s empowerment has been increasingly recognized as a strategy to improve physical health, its relationship with mental health remains less well understood.

**Objective:**

This study examined the association between women’s empowerment and mental health symptoms among ever-married women in Bangladesh.

**Methods:**

A cross-sectional analysis was conducted using data from the 2022 Bangladesh Demographic and Health Survey. Women’s empowerment was measured using the Survey-Based Women’s Empowerment (SWPER) Global index across 3 domains: attitudes toward violence, social independence, and decision-making. Associations between empowerment domains and depressive symptoms, anxiety, and any mental health symptoms were assessed using multivariable logistic regression.

**Results:**

Empowerment in the attitudes toward violence domain was significantly associated with mental health outcomes. Women with low empowerment in this domain had higher odds of depression (adjusted odds ratio [aOR] 1.81, 95% CI 1.16-2.81), anxiety (aOR 1.46, 95% CI 1.10-1.94), and any mental health symptoms (aOR 1.63, 95% CI 1.27-2.10). No significant associations were found for the social independence or decision-making domains.

**Conclusions:**

Higher empowerment in the “attitudes toward violence” domain was associated with a lower prevalence of common mental health symptoms, suggesting that addressing gender-based violence norms may improve women’s mental health in Bangladesh.

## Introduction

Mental health disorders are an increasingly critical global health concern, accounting for an estimated 17.2% of disability-adjusted life years (DALYs) in 2021 [1]. The burden is disproportionately high in low- and middle-income countries (LMICs), where limited health care access, stigma, and economic barriers restrict both prevention and treatment [2]. Women in LMICs experience a disproportionate burden of common mental disorders because of intersecting social, economic, and gender-related disadvantages, including poverty, limited educational and employment opportunities, restricted decision-making autonomy, gender discrimination, social isolation, intimate partner violence (IPV), and inadequate access to mental health services [2-4]. In South Asia, these structural inequities collectively contribute to depression, anxiety, and other common mental disorders among women [4]. Although IPV is a well-established determinant of women’s mental health, it represents only 1 pathway through which gender inequality may influence psychological well-being [5].

Women’s empowerment has emerged as an important social determinant of health and is recognized as a key component of Sustainable Development Goal (SDG) 5, which seeks to achieve gender equality and empower all women and girls [6]. Women’s empowerment is a multidimensional construct encompassing access to resources, decision-making autonomy, freedom of movement, social independence, and the ability to challenge inequitable gender norms [7]. Greater empowerment has been associated with improved reproductive, maternal, and child health outcomes, increased health care use, and enhanced overall well-being [8]. Emerging evidence also suggests that women’s empowerment may influence mental health by increasing autonomy, self-efficacy, and social support while reducing exposure to adverse social and gender-related stressors [9].

Although an increasing number of studies have examined the relationship between women’s empowerment and mental health, findings remain inconsistent across countries and empowerment domains. Some studies have reported that greater empowerment is associated with lower risks of depression and anxiety, whereas others have found weak or domain-specific associations [10-12]. These inconsistencies may partly reflect differences in the conceptualization and measurement of empowerment, as well as variation in sociocultural contexts. Consequently, examining empowerment as a multidimensional construct may provide a more comprehensive understanding of its relationship with women’s mental health.

In Bangladesh, these structural disadvantages are particularly salient. The country has made substantial progress in women’s education and maternal health, yet persistent gender inequalities continue to affect women’s social and economic opportunities [13]. Women continue to face barriers related to employment, household decision-making, child marriage, and gender-based violence [13]. Social norms that justify violence against women remain prevalent in many communities, and IPV remains widespread, with 23.2% of women aged 15 to 49 years reporting physical or sexual violence in the past year [14]. These structural disadvantages may adversely affect both women’s empowerment and their mental and physical health. Previous studies conducted in Bangladesh have primarily reported that lower autonomy, limited decision-making power, and exposure to gender-based violence are associated with poorer mental health outcomes [12,15-18]. However, these studies generally focused on specific dimensions of empowerment or selected populations, limiting our understanding of how multidimensional empowerment influences mental health at the national level.

Various indices have been used to measure empowerment, including the Women’s Empowerment in Agriculture Index [19], the Gender Development Index [20], and the Gender Inequality Index [21]. Although useful at national and regional levels, these tools offer limited applicability for subnational or subgroup analyses. To address this gap, the Survey-Based Women’s Empowerment (SWPER) Global index was developed in 2017 using Demographic and Health Survey (DHS) data from 34 African countries [22]. The index identifies 3 domains of empowerment—attitudes toward violence, social independence, and decision-making—and has since been adapted as the SWPER Global index for use across world regions [22,23]. The SWPER Global index provides a standardized multidimensional measure of women’s empowerment across these 3 domains [23]. Although the relationship between IPV victimization and women’s mental health has been extensively documented, considerably less is known about whether women’s attitudes toward the acceptability of violence—measured as an empowerment domain rather than as exposure to IPV—are independently associated with mental health [24,25]. Understanding this relationship may provide new insights into how gender norms and women’s empowerment influence psychological well-being beyond direct experiences of violence.

Evidence on women’s empowerment and mental health in South Asia remains limited. A recent study in Nepal, using the SWPER Global index, found that social independence was protective against depression and anxiety, whereas decision-making was associated with increased risk [26]. Previous studies across various contexts have reported mixed effects; some have identified empowerment—particularly autonomy and agency—as protective against mental health symptoms [19,27-29], while others have reported detrimental effects linked to specific domains such as decision-making [11,26]. Such findings highlight the complexity and context-specific nature of the relationship between empowerment and mental health.

Despite growing evidence linking women’s empowerment and mental health, important knowledge gaps remain. Few nationally representative studies have examined multidimensional women’s empowerment in relation to mental health in Bangladesh, and the relative contributions of different empowerment domains remain unclear, particularly the role of attitudes toward the acceptability of violence. Given persistent gender inequalities and limited mental health resources in Bangladesh, understanding these relationships is essential for informing gender-responsive public health interventions. Therefore, this study examined the associations between the 3 SWPER Global empowerment domains and symptoms of depression, anxiety, and overall mental health among ever-married women in Bangladesh. We hypothesize that lower levels of empowerment across the attitudes toward violence, social independence, and decision-making domains would be associated with greater odds of adverse mental health outcomes.

## Methods

### Data Source

This study analyzed secondary data from the women’s individual recode (IR) file of the nationally representative 2022 Bangladesh Demographic and Health Survey (BDHS). The BDHS was conducted in urban and rural areas across all 8 administrative divisions of Bangladesh between June and December 2022. It used a 2-stage stratified cluster sampling design based on the 2011 national census. In the first stage, 675 enumeration areas were selected with probability proportional to size. In the second stage, an average of 45 households per cluster were selected systematically, yielding a sample of 30,330 households. Ever-married women aged 15 to 49 years who were usual residents of, or who stayed in, selected households on the night before the survey were eligible for the interview. Thirty of the 45 selected households in each cluster were randomly allocated to the long women’s questionnaire, which included the Patient Health Questionnaire–9 (PHQ-9) and Generalized Anxiety Disorder–7 (GAD-7) mental health modules. For this analysis, only women with complete data for SWPER Global empowerment indicators and mental health measures were included (N=17,027). Details of the BDHS methodology are available elsewhere [30].

### Exposure Variable

Women’s empowerment was the exposure variable, assessed using the SWPER Global index, which was constructed from 14 standardized DHS items available in the women’s IR file for married women. The index includes 3 domains: attitudes toward violence, social independence, and decision-making. The attitudes toward violence domain reflects women’s acceptance or rejection of justifications for wife beating; the social independence domain captures women’s education, age at marriage or first birth, age difference with the partner, and media exposure; and the decision-making domain reflects participation in household decisions. Domain scores were generated using principal component analysis and then categorized into tertiles (low, medium, and high) following established SWPER Global methodology [22,23].

### Outcome Variables

This study evaluated 3 mental health outcomes: depression, anxiety, and any mental health symptoms. Anxiety was assessed using the GAD-7 scale [31]. Participants reported the frequency of their symptoms on a scale from 0 (“never”) to 3 (“always”) over the preceding 2 weeks, with responses to the 7 questions aggregated to yield a total score between 0 and 21. Respondents were categorized as exhibiting anxiety symptoms if they achieved a GAD-7 score of ≥6. This cutoff was used for the present population-based analysis as a symptom-screening definition rather than a clinical diagnosis. Depression was assessed using the PHQ-9 [32]. Participants reported the occurrence of their symptoms on a scale from 0 (“never”) to 3 (“always”), with responses to the 9 questions aggregated to yield a cumulative score between 0 and 27. Respondents were categorized as exhibiting depressive symptoms if they achieved a PHQ-9 score of ≥10. Respondents were classified as exhibiting “any mental health symptoms” if they reported experiencing ≥1 symptom of anxiety and/or depression. This study categorized mental health symptoms according to methodologies established in prior research [26]. Although the PHQ-9 and GAD-7 are screening rather than diagnostic instruments, Bangladeshi validation studies have supported their reliability and validity in local samples, including evidence for unidimensionality and acceptable internal consistency [33].

### Covariates

This study included various sociodemographic covariates such as age, place of residence, division, highest level of education, number of children, wealth index, religion, the respondent’s employment status, the partner’s employment status, and the respondent’s BMI. These variables were selected a priori as potential confounders because they may be associated with both women’s empowerment and mental health. Religion was included to account for cultural and normative differences that may shape gender roles and attitudes toward women’s autonomy; partner employment status was included as a marker of household economic stability and financial stress; and BMI was included as an indicator of women’s physical and nutritional status, which may be linked to both psychological well-being and social disadvantage. Age was classified into 3 groups: 15 to 29 years, 30 to 39 years, and 40 to 49 years. Residence was classified as urban or rural according to country-specific criteria [30]. Bangladesh comprises 8 divisions: Barishal, Chattogram, Dhaka, Khulna, Mymensingh, Rajshahi, Rangpur, and Sylhet. Education level was classified according to the highest level of schooling completed: no education, primary, secondary, or higher education. Children were classified into 3 categories: 0 or 1 child, 2 children, and ≥3 children, according to population distribution. The wealth index was developed through principal component analysis, a statistical technique frequently used to create composite indicators of economic status derived from household assets and characteristics. The population was subsequently categorized into quintiles, from the poorest to the richest [30]. The respondent’s reported religion was categorized as either “Islam” or “Other.” The employment status of both the respondent and their partner was categorized as binary variables: employed or unemployed. BMI was classified according to World Health Organization (WHO) standards: underweight (<18.5 kg/m^2^), normal weight (18.5 kg/m^2^-24.9 kg/m^2^), overweight (25.0 kg/m^2^-29.9 kg/m^2^), and obese (≥30.0 kg/m^2^). This study categorized BMI into 2 groups: “normal” (18.5 kg/m^2^-24.9 kg/m^2^) and “underweight/overweight” (<18.5 kg/m^2^ or ≥25.0 kg/m^2^) to identify individuals outside the conventional “normal” range.

### Statistical Analysis

Descriptive statistics were used to summarize sample characteristics, with proportions estimated for categorical variables and means and SDs reported for continuous variables. The prevalence of anxiety, depression, and any mental health symptoms was calculated by sociodemographic characteristics and SWPER Global domains. Bivariate associations were evaluated using chi-square tests. Associations between empowerment levels and mental health outcomes were examined with bivariate and multivariate logistic regression; covariates with *P*<.25 in bivariate analyses were included in the multivariate models. Collinearity among covariates was assessed using variance inflation factors. Adjusted odds ratios (aORs) with 95% CIs were reported. Statistical significance was determined using an α level of .05, with all tests being 2-sided. Model fit was assessed using the Archer-Lemeshow *F*-adjusted goodness-of-fit test for survey-weighted logistic regression; a nonsignificant result indicated no evidence of lack of fit. All analyses were conducted in Stata (version 18.5; StataCorp) using survey commands (*svy*) to apply the BDHS sampling weights, strata, and primary sampling unit, thereby producing nationally representative estimates and valid SEs.

### Sensitivity Analyses

We re-estimated all associations using survey-weighted Poisson regression with a log link to obtain adjusted prevalence ratios (aPRs) and 95% CIs. These models included the same exposures and covariates as the primary logistic regression models and accounted for the BDHS sampling weights, stratification, and clustering. This analysis was undertaken because odds ratios (ORs) can overstate the magnitude of association when outcomes are common in cross-sectional studies [34].

### Ethical Considerations

The 2022 BDHS protocol was reviewed and approved by the Institutional Review Board of Inner City Fund (ICF) International and the Bangladesh Medical Research Council (BMRC). The publicly available BDHS documentation does not report the corresponding approval numbers. Before participation, all respondents provided informed consent to take part in the survey. The present study was a secondary analysis of deidentified BDHS data obtained through the DHS program. The analytic dataset contained no direct personal identifiers, and the research team had no access to information that could identify individual participants. Therefore, no additional participant contact, recruitment, or consent was required for this analysis. No compensation was provided to participants for this secondary analysis. The manuscript and supplementary materials contain no identifiable participant information, including names, photographs, addresses, or other potentially identifying details. Detailed ethical aspects and consent forms are available on the BDHS website and in the report.

## Results

This study included a total of 17,027 respondents, representing a population of ever-married women aged 15 to 49 years who responded to the questions required for calculating the SWPER Global index and assessing mental health in the 2022 BDHS. Table 1 presents a summary of the sociodemographic characteristics of the respondents included in the study.

**Table 1.** Sociodemographic characteristics of included 2022 Bangladesh Demographic and Health Survey respondents (N=17,027).

Sociodemographic characteristics	Participants, n (%)
Age (y), mean (SD)	32.82 (8.40)
Age group (y), n (%)	
15-29	6492 (38.1)
30-39	6286 (36.9)
40-49	4249 (25)
Residence type, n (%)	
Urban	5920 (34.8)
Rural	11,107 (65.2)
Division, n (%)	
Barishal	1819 (10.7)
Chattogram	2545 (14.9)
Dhaka	2560 (15)
Khulna	2229 (13.1)
Mymensingh	1837 (10.8)
Rajshahi	2172 (12.8)
Rangpur	2082 (12.2)
Sylhet	1783 (10.5)
Highest education level, n (%)	
No education	2354 (13.8)
Primary	4684 (27.5)
Secondary	7677 (45.1)
Higher	2312 (13.6)
Number of children, mean (SD)	2.25 (1.13)
Number of children grouped, n (%)	
0-1	4539 (26.6)
2	6707 (39.4)
≥3	5781 (34)
Household wealth index, n (%)	
Poorest	3089 (18.1)
Poorer	3361 (19.7)
Middle	3384 (19.9)
Richer	3518 (20.7)
Richest	3675 (21.6)
Religion, n (%)	
Islam	15,229 (89.4)
Other	1798 (10.6)
Respondent working, n (%)	
Yes	5402 (31.7)
No	11,625 (68.3)
Husband or partner working, n (%)	
Yes	16,505 (97)
No	506 (3)
BMI of respondents (kg/m^2^),^a^ mean (SD)	23.97 (4.31)
BMI group^a^, n (%)	
Normal (18.5 kg/m^2^-24.9 kg/m^2^)	4462 (52.5)
Abnormal (<18.5 kg/m^2^ or ≥25.0 kg/m^2^)	4029 (47.5)

a Some sociodemographic characteristics were not reported by all respondents; therefore, the total for these variables may not be equal to the sample size (N=17,027).

Mental health outcomes were stratified by sociodemographic characteristics and summarized in Table 2. Across the sample, 4.9% of respondents were classified as having depression, 19.5% as having anxiety, and 31.9% as having at least 1 mental health symptom in the 2022 BDHS. Education was inversely associated with the prevalence of anxiety, depression, and any mental health symptoms, with lower levels of education associated with a higher prevalence of mental health symptoms. Similarly, the household wealth index was inversely associated with mental health outcomes, with poorer individuals more likely to experience depression, anxiety, and any mental health symptoms. Being underweight or overweight was associated with a higher prevalence of anxiety, depression, and any mental health symptoms (Table 2).

**Table 2.** Unweighted frequencies and weighted prevalence of depression, anxiety, and any mental health symptoms (MHSs) by women’s empowerment domain.^a^

Empowerment domains or categories	Depression (n=830), n (weighted %)	*P* value	Anxiety (n=3269), n (weighted %)	*P* value	Any MHSs (n=5417), n (weighted %)	*P* value
SWPER^b^—attitudes toward violence		.001		<.001		<.001
Low	48 (7.6)		174 (27.1)		262 (41.7)	
Medium	107 (5.8)		421 (23.5)		695 (39.9)	
High	675 (4.4)		2674 (18.2)		4460 (30)	
SWPER—social independence		.01		<.001		<.001
Low	425 (5.3)		1712 (21.3)		2646 (33)	
Medium	291 (4.3)		1120 (17.5)		1973 (30.8)	
High	114 (3.8)		437 (16.1)		798 (28.4)	
SWPER—decision-making		.60		.048		.007
Low	96 (4.4)		383 (16.9)		653 (29.4)	
Medium	194 (4.5)		834 (18.8)		1518 (33.8)	
High	540 (4.9)		2052 (19.7)		3246 (31)	

a Frequencies are unweighted numbers of women with depression, anxiety, or at least 1 mental health symptom. Percentages are weighted estimates accounting for the 2022 Bangladesh Demographic and Health Survey sampling weights, stratification, and clustering. *P* values were calculated using design-based Pearson chi-square tests.

b SWPER: Survey-Based Women’s Empowerment.

The distribution of respondents’ empowerment levels varied across the 3 SWPER Global domains. Most women had high empowerment in the “attitudes toward violence” (86.2%) and decision-making (60.7%) domains, whereas nearly half had low empowerment in the social independence domain (46.3%). Overall, higher empowerment across the 3 domains was more common among women who lived in urban areas, had higher educational attainment, and belonged to wealthier households. Detailed distributions of empowerment levels according to sociodemographic characteristics are presented in Tables S1A to S1C in Multimedia Appendix 1.

**Figure 1.**
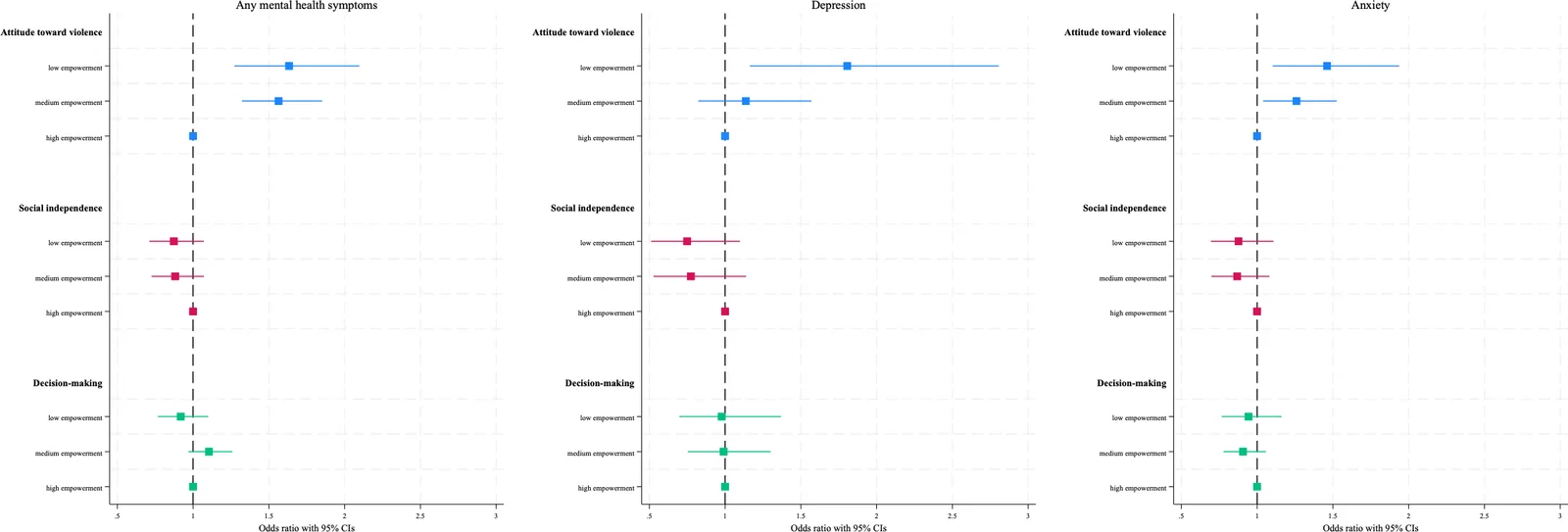
Adjusted odds ratios presenting the associations between women’s empowerment domains and mental health symptom prevalence. Odds ratios were adjusted for age, residence type, division, education level, number of children, religion, employment status (respondent and partner), and BMI.

Unadjusted ORs for the associations between women’s empowerment domains and mental health outcomes are summarized in Table 3. Figure 1 presents graphical representations of aORs after adjustment for covariates including age, residence type, division, education level, number of children, wealth index, religion, work status, and BMI. Results indicated a significant association between mental health outcomes and empowerment in the “attitudes toward violence” domain. Women with low empowerment in this domain had higher odds of depression (aOR 1.81, 95% CI 1.16-2.81), anxiety (aOR 1.46, 95% CI 1.10-1.94), and any mental health symptoms (aOR 1.63, 95% CI 1.27-2.10). Medium empowerment in this domain was also associated with higher odds of anxiety (aOR 1.26, 95% CI 1.04-1.53) and any mental health symptoms (aOR 1.56, 95% CI 1.32-1.85). No significant association was observed between empowerment in the social independence or decision-making domains and mental health outcomes in this study. The Archer-Lemeshow *F*-adjusted goodness-of-fit test indicated no evidence of lack of fit for all final models (*P*>.05), except for the model examining depression in relation to the “Attitude to Violence” domain (*P*=.03).

**Table 3.** Unadjusted associations of SWPER (Survey-Based Women’s Empowerment) Global index domains and mental health symptoms (MHSs) among women in Bangladesh^a^.

Empowerment domains or categories	Depression, OR^b^ (95% CI)	Anxiety, OR (95% CI)	Any MHSs, OR (95% CI)
SWPER—attitudes toward violence			
Low	2.05 (1.46-2.87)^c^	1.78 (1.44-2.22)^c^	1.72 (1.41-2.11)^c^
Medium	1.36 (1.08-1.71)^d^	1.41 (1.22-1.64)^c^	1.58 (1.40-1.80)^c^
High	1.00	1.00	1.00
SWPER—social independence			
Low	1.39 (1.07-1.79)^d^	1.40 (1.23-1.60)^c^	1.24 (1.11-1.39)^c^
Medium	1.13 (0.87-1.45)	1.09 (0.95-1.25)	1.12 (1.00-1.25)^e^
High	1.00	1.00	1.00
SWPER—decision-making			
Low	0.89 (0.69-1.19)	0.83 (0.71-0.98)^e^	0.93 (0.81-1.06)
Medium	0.92 (0.74-1.14)	0.94 (0.84-1.05)	1.14 (1.04-1.25)^d^
High	1.00	1.00	1.00

a Statistical significance was determined using 2-sided tests with an α of .05. Empowerment levels were calculated using the SWPER Global index and categorized using the original studies’ methodologies [22,23].

b OR: odds ratio; unadjusted ORs are based on bivariate logistic regression models.

c *P*<.001.

d *P*<.01.

e *P*<.05.

Sensitivity analyses using survey-weighted Poisson regression produced aPRs that were consistent in direction and statistical significance with the primary logistic regression findings (Tables S2A-S2C in Multimedia Appendix 1).

## Discussion

### Principal Findings

This study found a significant association between women’s empowerment in the “attitudes toward violence” domain and mental health outcomes among Bangladeshi women, whereas no significant association was observed for the social independence or decision-making domains. These findings indicate that the relationship between empowerment and mental health is multidimensional and may vary according to the specific dimension of empowerment being assessed.

The present findings are broadly consistent with evidence from neighboring South Asian countries, although the patterns are not uniform across settings. A recent nationally representative study from Nepal reported that greater social independence was associated with lower odds of anxiety and depression, whereas higher decision-making empowerment was associated with increased odds of these symptoms [26]. Similarly, prospective evidence from rural India suggests that the relationship between women’s agency and mental distress is complex and context-dependent rather than uniformly protective [11]. In Pakistan, population-based research has shown that IPV is strongly associated with poorer mental health among married women, highlighting the broader regional importance of violence-related gender norms for women’s psychological well-being [35].

### Attitudes Toward Violence

Women with lower empowerment in the “attitudes toward violence” domain—those less likely to reject justifications for IPV—had significantly higher odds of depression, anxiety, and any mental health symptoms, even after adjusting for confounders. Although the observed aORs were modest in magnitude, they may still be important at the population level because even small shifts in common mental health symptoms can translate into substantial public health impacts in a large population. The findings therefore suggest that attitudes toward violence may be a meaningful but not the sole determinant of women’s mental health in Bangladesh. This finding is consistent with broader literature showing that IPV and tolerance of violence are closely linked to women’s psychological distress [36,37]. In Bangladesh, previous studies have shown that IPV is associated with depression and that women exposed to violence may face substantial barriers to seeking mental health support [38-40]. Studies in South Asia have also demonstrated that women experiencing IPV report poorer psychological well-being, while attitudinal rejection of IPV can promote resilience and access to social support and services [11,26,35].

Less attention has been paid to the role of women’s attitudes toward IPV, as distinct from victimization itself, in shaping mental health outcomes. Recent evidence from Nepal found no significant association between the SWPER Global “attitudes toward violence” domain and mental health symptom prevalence [12], in contrast to the protective effects found in this Bangladeshi sample. The observed benefits in Bangladesh may reflect context-specific pathways. One possible explanation is that women who reject IPV may also differ in other unmeasured ways that are linked to better mental health, although the present cross-sectional data cannot establish such pathways. Given that 23.2% of women in Bangladesh have experienced IPV [8], attitudinal rejection may be a critical focus for both empowerment and mental health interventions.

Taken together, these findings suggest that attitudes toward IPV may capture an important dimension of internalized gender norms that is especially relevant to mental health in Bangladesh. Women who reject the acceptability of IPV may be more likely to recognize abuse, seek support, and resist harmful relationship dynamics, whereas acceptance of IPV may reflect deeper social normalization of violence and reduced psychosocial agency. In a setting where IPV remains prevalent, this domain may therefore be particularly salient for understanding women’s mental health vulnerability.

### Social Independence

The association between the “social independence” domain and mental health symptoms was not statistically significant after adjustment for confounders, although the unadjusted analyses suggested a modest protective pattern. The absence of a statistically significant association for social independence does not necessarily indicate that no relationship exists. Rather, it may suggest that any effect is small, context-dependent, or not fully captured by the current cross-sectional measures.

This mixed pattern aligns with existing literature reporting both protective and adverse effects of social independence–related empowerment on women’s mental health. In Nepal, higher social independence was associated with lower odds of both anxiety and depression [26]. In Bangladesh, related research among female garment workers suggests that empowerment-related resource mobilization may contribute to lower depressive symptoms and better self-esteem [27]. At the same time, evidence from India indicated that women who were more comfortable speaking up in public reported greater mental distress, possibly because of social backlash and increased visibility within conservative communities [11].

These divergent findings underscore the context-dependent and multidimensional nature of social independence as an empowerment construct. Although greater social independence may be associated with improved access to resources, mobility, and support, it may also coincide with increased scrutiny, interpersonal conflict, or other social pressures in more conservative settings. Because this study is cross-sectional, these patterns should be interpreted cautiously and should not be taken to indicate causal pathways. It is also possible that more socially independent women are more likely to recognize or report psychological symptoms, which could influence observed associations. Further longitudinal and qualitative research is needed to clarify how social independence relates to women’s mental health across different contexts.

### Decision-Making

This study found no statistically significant association between women’s empowerment in the “decision-making” domain and mental health symptoms. The absence of a statistically significant association for decision-making does not necessarily imply that there is no relationship. Instead, it may reflect a small, context-dependent effect or a pattern that is not well captured by the current cross-sectional design and measurement approach. However, this finding is consistent with the broader literature showing that household decision-making does not have a uniform relationship with women’s mental health. For example, higher decision-making empowerment has been associated with increased odds of anxiety and depression in Nepal [26], whereas studies from Mozambique [41] and other sub-Saharan African settings [28,42] have reported protective associations. Evidence from India further suggests that greater decision-making autonomy may be accompanied by psychological strain when women assume nontraditional roles in patriarchal settings where such changes are not fully supported by families or communities [11,26].

The lack of a significant relationship in our study should be interpreted cautiously. Because this study is cross-sectional, it does not allow causal inference. One possible explanation is that decision-making autonomy may be associated with both potential benefits and potential burdens: although it may increase women’s control over household matters, it may also coincide with greater responsibility, interpersonal tension, or social and familial resistance. These findings suggest that the relationship between household decision-making and mental health may be context-dependent, and that instrumental forms of empowerment do not necessarily correspond to better psychological well-being in all settings.

### Mental Health and Empowerment Patterns Across Sociodemographic Groups

In our sample, depression affected a smaller proportion of respondents (4.9%) than that reported in both the 2019 National Mental Health Survey in Bangladesh (7.9%) [43] and the 2021 Global Burden of Disease (GBD) estimate for women aged 15 to 49 years (8.3%). In contrast, anxiety prevalence (19.5% in this study) was notably higher than the estimates from both sources (5.4% and 5.5%, respectively) [43].

Several methodological differences may help explain these discrepancies. The National Mental Health Survey used stricter diagnostic procedures, including assessment by trained research psychiatrists, whereas the present study relied on screening instruments included in the BDHS. The GBD estimates may also reflect older underlying data, and differences in instruments, symptom thresholds, and case definitions across studies may further affect prevalence estimates.

### Policy Implications

The present findings have several practical implications for mental health and gender equity programming in Bangladesh. Because the attitudes toward violence domain was the only empowerment dimension consistently associated with depression, anxiety, and overall mental health symptoms, interventions that aim to reduce the social acceptability of IPV may have benefits beyond violence prevention alone. Mental health providers, community health workers, and frontline practitioners could integrate brief screening for violence-related norms, psychological distress, and support needs into routine services for women, particularly in primary care and reproductive health settings. Public health programs may also benefit from combining mental health promotion with community-based efforts to challenge harmful gender norms, strengthen referral pathways, and improve access to psychosocial support for women at risk of both violence and poor mental health.

These findings also suggest several directions for future research and policy. Longitudinal studies are needed to better understand the causal pathways linking different empowerment domains and mental health over time. Further qualitative and mixed methods research could help clarify why attitudes toward violence appear more strongly related to mental health than social independence or decision-making in this setting. At the policy level, integrating women’s empowerment indicators into mental health surveillance and embedding gender-transformative approaches within national mental health and violence prevention strategies may help translate these findings into more effective and contextually appropriate interventions.

### Strengths and Limitations

This study has several limitations. First, all data were self-reported, raising the possibility of reporting bias—especially on sensitive topics such as mental health and women’s empowerment—due to stigma or gender norms in Bangladesh. Highly empowered women may also be more likely to recognize and disclose mental health symptoms, potentially inflating observed associations. The BDHS used screening instruments rather than clinical diagnostic tools, which may underestimate the true burden of mental health symptoms and limit the scope of assessed conditions. Although the SWPER Global index is a validated tool, it may not fully capture the cultural and contextual nuances of empowerment. Despite adjusting for key sociodemographic factors, unmeasured confounders—including childhood trauma, extended family dynamics, or access to mental health services—may influence results. We included religion, partner employment status, and BMI as contextual covariates to better account for the cultural environment, household economic conditions, and women’s physical health, all of which may plausibly influence both empowerment and mental health. Although these were not the primary variables of interest, their inclusion was intended to reduce residual confounding and improve interpretation of the observed associations.

The main strengths of this study include its use of a large, nationally representative BDHS sample, which enhances the generalizability of the findings to Bangladeshi women. The use of the SWPER Global index also allowed separate examination of 3 distinct dimensions of empowerment—attitudes toward violence, social independence, and decision-making—rather than treating empowerment as a single construct, which improves the specificity of the findings. In addition, the study used standardized screening measures for anxiety and depression and adjusted for several relevant sociodemographic and contextual covariates, which strengthens internal validity and supports a more nuanced interpretation of the observed associations. Finally, this research is among the first to systematically explore the association between women’s empowerment and mental health symptom prevalence in Bangladesh, addressing an important gap in the literature.

### Conclusions

In this nationally representative study of Bangladeshi women, only the SWPER Global domain of attitudes toward violence was independently associated with lower prevalence of mental health symptoms after adjusting for sociodemographic factors. Women who rejected justifications for IPV reported lower prevalence of depression, anxiety, and overall mental health symptoms. The lack of association for the social independence and decision-making domains, coupled with inconsistencies across the literature, underscores the need for contextually tailored interventions. These findings highlight the importance of integrating mental health support and targeting attitudes toward the acceptance of IPV and harmful gender norms within women’s empowerment initiatives in Bangladesh. In practice, these results support the inclusion of violence-norm screening, community awareness activities, and culturally responsive psychosocial support within women’s health and primary care services. Future research should further investigate the mechanisms linking empowerment domains and mental health to guide more targeted interventions for Bangladeshi women.

## Supplementary material

10.2196/85839 Multimedia Appendix 1 Additional analyses.

## Abbreviations

aOR: adjusted odds ratio
aPR: adjusted prevalence ratio
BDHS: Bangladesh Demographic and Health Survey
BMRC: Bangladesh Medical Research Council
DALY: disability-adjusted life year
DHS: Demographic and Health Survey
GAD-7: Generalized Anxiety Disorder–7
GBD: Global Burden of Disease
ICF: Inner City Fund
IPV: intimate partner violence
IR: individual recode
LMIC: low- and middle-income country
OR: odds ratio
PHQ-9: Patient Health Questionnaire–9
SDG: Sustainable Development Goal
SWPER: Survey-Based Women’s Empowerment
WHO: World Health Organization
