# Supplementary material for: Linking Women’s Empowerment to Mental Health Symptoms: Population-Based Study
Source: JMIR Public Health Surveill. 2026 Sep 8;12:e85839. doi: 10.2196/85839 (PMC13552646; doi:10.2196/85839)
Supplement: Supplementary file 1 [file publichealth-v12-e85839-s001.docx]

**Supplementary Tables**

**Table 1a**. Level of women’s empowerment in the “Attitudes Towards Violence” domain of the SWPER Global index by sociodemographic characteristics.

|  | Total | Low | Medium | High |
| --- | --- | --- | --- | --- |
| N (%) | 17,027 (100%) | 607 (3.6%) | 1,735 (10.2%) | 14,685 (86.2%) |
| Age |  |  |  |  |
| 15-29 yrs | 6,492 (38.1%) | 197 (32.5%) | 602 (34.7%) | 5,693 (38.8%) |
| 30-39 yrs | 6,286 (36.9%) | 229 (37.7%) | 659 (38%) | 5,398 (36.8%) |
| 40-49 yrs | 4,249 (25%) | 181 (29.8%) | 474 (27.3%) | 3,594 (24.5%) |
| Residence Type |  |  |  |  |
| Urban | 5,920 (34.8%) | 143 (23.6%) | 536 (30.9%) | 5,241 (35.7%) |
| Rural | 11,107 (65.2%) | 464 (76.4%) | 1,199 (69.1%) | 9,444 (64.3%) |
| Division |  |  |  |  |
| Barishal | 1,819 (10.7%) | 46 (7.6%) | 169 (9.7%) | 1,604 (10.9%) |
| Chattogram | 2,545 (14.9%) | 80 (13.2%) | 246 (14.2%) | 2,219 (15.1%) |
| Dhaka | 2,560 (15%) | 106 (17.5%) | 308 (17.8%) | 2,146 (14.6%) |
| Khulna | 2,229 (13.1%) | 112 (18.5%) | 265 (15.3%) | 1,852 (12.6%) |
| Mymensingh | 1,837 (10.8%) | 71 (11.7%) | 159 (9.2%) | 1,607 (10.9%) |
| Rajshahi | 2,172 (12.8%) | 84 (13.8%) | 264 (15.2%) | 1,824 (12.4%) |
| Rangpur | 2,082 (12.2%) | 49 (8.1%) | 196 (11.3%) | 1,837 (12.5%) |
| Sylhet | 1,783 (10.5%) | 59 (9.7%) | 128 (7.4%) | 1,596 (10.9%) |
| Highest Education |  |  |  |  |
| No education | 2,354 (13.8%) | 130 (21.4%) | 297 (17.1%) | 1,927 (13.1%) |
| Primary | 4,684 (27.5%) | 222 (36.6%) | 539 (31.1%) | 3,923 (26.7%) |
| Secondary | 7,677 (45.1%) | 232 (38.2%) | 771 (44.4%) | 6,674 (45.4%) |
| Higher | 2,312 (13.6%) | 23 (3.8%) | 128 (7.4%) | 2,161 (14.7%) |
| Number of Children |  |  |  |  |
| 0-1 child | 4,539 (26.7%) | 129 (21.3%) | 407 (23.5%) | 4,003 (27.3%) |
| 2 children | 6,707 (39.4%) | 223 (36.7%) | 673 (38.8%) | 5,811 (39.6%) |
| 3+ children | 5,781 (34%) | 255 (42%) | 655 (37.8%) | 4,871 (33.2%) |
| Wealth Index |  |  |  |  |
| Poorest | 3,089 (18.1%) | 163 (26.9%) | 372 (21.4%) | 2,554 (17.4%) |
| Poorer | 3,361 (19.7%) | 140 (23.1%) | 375 (21.6%) | 2,846 (19.4%) |
| Middle | 3,384 (19.9%) | 113 (18.6%) | 367 (21.2%) | 2,904 (19.8%) |
| Richer | 3,518 (20.7%) | 118 (19.4%) | 342 (19.7%) | 3,058 (20.8%) |
| Richest | 3,675 (21.6%) | 73 (12%) | 279 (16.1%) | 3,323 (22.6%) |
| Religion |  |  |  |  |
| Islam | 15,299 (89.4%) | 575 (94.7%) | 1,591 (91.7%) | 13,063 (89%) |
| Other | 1,798 (10.6%) | 32 (5.3%) | 144 (8.3%) | 1,622 (11%) |
| Respondent Working |  |  |  |  |
| No | 11,625 (68.3%) | 394 (64.9%) | 1,146 (66.1%) | 10,085 (68.7%) |
| Yes | 5,402 (31.7%) | 213 (35.1%) | 589 (33.9%) | 4,600 (31.3%) |
| Husband/Partner Working |  |  |  |  |
| No | 506 (3%) | 13 (2.1%) | 43 (2.5%) | 450 (3.1%) |
| Yes | 16,505 (97%) | 592 (97.9%) | 1,691 (97.5%) | 14,222 (96.9%) |
| BMI |  |  |  |  |
| Normal | 4,462 (52.5%) | 164 (54.3%) | 449 (51.5%) | 3,849 (52.6%) |
| Underweight or Overweight | 4,029 (47.5%) | 138 (45.7%) | 423 (48.5%) | 3,468 (47.4%) |
| Any Mental Health Symptoms |  |  |  |  |
| None | 11,609 (68.2%) | 345 (56.8%) | 1,039 (59.9%) | 10,225 (69.6%) |
| At least one symptom | 5,417 (31.8%) | 262 (43.2%) | 695 (40.1%) | 4,460 (30.4%) |
| Anxiety |  |  |  |  |
| No Anxiety | 13,758 (80.8%) | 433 (71.3%) | 1,314 (75.7%) | 12,011 (81.8%) |
| Anxiety | 3,269 (19.2%) | 174 (28.7%) | 421 (24.3%) | 2,674 (18.2%) |
| Depression |  |  |  |  |
| No Depression | 16,197 (95.1%) | 559 (92.1%) | 1,628 (93.8%) | 14,010 (95.4%) |
| Depression | 830 (4.9%) | 48 (7.9%) | 107 (6.2%) | 675 (4.6%) |

*^a^ Empowerment levels were calculated using the SWPER Global index and categorized using the original studies’ methodologies [12, 13]. Note: Some sociodemographic characteristics were not reported by every respondent; therefore, the total for these groups may not be equal to the total N = 17,027. Abbreviations: SWPER – Survey-based Women’s emPowERment; BMI – body mass index; GAD – Generalized Anxiety Disorder.*

**Table 1b**. Level of women’s empowerment in the “Social Independence” domain of the SWPER Global index by sociodemographic characteristics.

|  | Total | Low | Medium | High |
| --- | --- | --- | --- | --- |
| N (%) | 17,027 (100%) | 7,883 (46.3%) | 6,455 (37.9%) | 2,689 (15.8%) |
| Age |  |  |  |  |
| 15-29 yrs | 6,492 (38.1%) | 2,586 (32.8%) | 2,961 (45.9%) | 945 (35.1%) |
| 30-39 yrs | 6,286 (36.9%) | 2,794 (35.4%) | 2,343 (36.3%) | 1,149 (42.7%) |
| 40-49 yrs | 4,249 (25%) | 2,503 (31.8%) | 1,151 (17.8%) | 595 (22.1%) |
| Residence Type |  |  |  |  |
| Urban | 5,920 (34.8%) | 2,351 (29.8%) | 2,220 (34.4%) | 1,349 (50.2%) |
| Rural | 11,107 (65.2%) | 5,532 (70.2%) | 4,235 (65.6%) | 1,340 (49.8%) |
| Division |  |  |  |  |
| Barishal | 1,819 (10.7%) | 805 (10.2%) | 712 (11%) | 302 (11.2%) |
| Chattogram | 2,545 (14.9%) | 980 (12.4%) | 1,157 (17.9%) | 408 (15.2%) |
| Dhaka | 2,560 (15%) | 1,141 (14.5%) | 961 (14.9%) | 458 (17%) |
| Khulna | 2,229 (13.1%) | 1,163 (14.8%) | 760 (11.8%) | 306 (11.4%) |
| Mymensingh | 1,837 (10.8%) | 878 (11.1%) | 704 (10.9%) | 255 (9.5%) |
| Rajshahi | 2,172 (12.8%) | 1,165 (14.8%) | 714 (11.1%) | 293 (10.9%) |
| Rangpur | 2,082 (12.2%) | 1,115 (14.1%) | 690 (10.7%) | 277 (10.3%) |
| Sylhet | 1,783 (10.5%) | 636 (8.1%) | 757 (11.7%) | 390 (14.5%) |
| Highest Education |  |  |  |  |
| No education | 2,354 (13.8%) | 1,969 (25%) | 337 (5.2%) | 48 (1.8%) |
| Primary | 4,684 (27.5%) | 3,078 (39%) | 1,403 (21.7%) | 203 (7.5%) |
| Secondary | 7,677 (45.1%) | 2,759 (35%) | 3,978 (61.6%) | 940 (35%) |
| Higher | 2,312 (13.6%) | 77 (1%) | 737 (11.4%) | 1,498 (55.7%) |
| Number of Children |  |  |  |  |
| 0-1 child | 4,539 (26.7%) | 1,282 (16.3%) | 2,001 (31%) | 1,256 (46.7%) |
| 2 children | 6,707 (39.4%) | 2,924 (37.1%) | 2,693 (41.7%) | 1,090 (40.5%) |
| 3+ children | 5,781 (34%) | 3,677 (46.6%) | 1,761 (27.3%) | 343 (12.8%) |
| Wealth Index |  |  |  |  |
| Poorest | 3,089 (18.1%) | 1,780 (22.6%) | 1,111 (17.2%) | 198 (7.4%) |
| Poorer | 3,361 (19.7%) | 1,796 (22.8%) | 1,254 (19.4%) | 311 (11.6%) |
| Middle | 3,384 (19.9%) | 1,661 (21.1%) | 1,304 (20.2%) | 419 (15.6%) |
| Richer | 3,518 (20.7%) | 1,546 (19.6%) | 1,406 (21.8%) | 566 (21%) |
| Richest | 3,675 (21.6%) | 1,100 (14%) | 1,380 (21.4%) | 1,195 (44.4%) |
| Religion |  |  |  |  |
| Islam | 15,299 (89.4%) | 7,213 (91.5%) | 5,758 (89.2%) | 2,258 (84%) |
| Other | 1,798 (10.6%) | 670 (8.5%) | 697 (10.8%) | 431 (16%) |
| Respondent Working |  |  |  |  |
| No | 11,625 (68.3%) | 5,035 (63.9%) | 4,688 (72.6%) | 1,902 (70.7%) |
| Yes | 5,402 (31.7%) | 2,848 (36.1%) | 1,767 (27.4%) | 787 (29.3%) |
| Husband/Partner Working |  |  |  |  |
| No | 506 (3%) | 276 (3.5%) | 149 (2.3%) | 81 (3%) |
| Yes | 16,505 (97%) | 7,598 (96.5%) | 6,301 (97.7%) | 2,606 (97%) |
| BMI |  |  |  |  |
| Normal | 4,462 (52.5%) | 2,080 (53.6%) | 1,696 (52.5%) | 686 (49.9%) |
| Underweight or Overweight | 4,029 (47.5%) | 1,803 (46.4%) | 1,536 (47.5%) | 690 (50.1%) |
| Any Mental Health Symptoms |  |  |  |  |
| None | 11,609 (68.2%) | 5,237 (66.4%) | 4,481 (69.4%) | 1,891 (70.3%) |
| At least one symptom | 5,417 (31.8%) | 2,646 (33.6%) | 1,973 (30.6%) | 798 (29.7%) |
| Anxiety |  |  |  |  |
| No Anxiety | 13,758 (80.8%) | 6,171 (78.3%) | 5,335 (82.6%) | 2,252 (83.7%) |
| Anxiety | 3,269 (19.2%) | 1,712 (21.7%) | 1,120 (17.4%) | 437 (16.3%) |
| Depression |  |  |  |  |
| No Depression | 16,197 (95.1%) | 7,458 (94.6%) | 6,164 (95.5%) | 2,575 (95.8%) |
| Depression | 830 (4.9%) | 425 (5.4%) | 291 (4.5%) | 114 (4.2%) |

*^a^ Empowerment levels were calculated using the SWPER Global index and categorized using the original studies’ methodologies [12, 13]. Note: Some sociodemographic characteristics were not reported by every respondent; therefore, the total for these groups may not be equal to the total N = 17,027 Abbreviations: SWPER – Survey-based Women’s emPowERment; BMI – body mass index; GAD – Generalized Anxiety Disorder.*

**Table 1c**. Level of women’s empowerment in the “Decision-Making” domain of the SWPER Global index by sociodemographic characteristics.

|  | Total | Low | Medium | High |
| --- | --- | --- | --- | --- |
| N (%) | 17,027 (100%) | 2,284 (13.4%) | 4,412 (25.9%) | 10,331 (60.7%) |
| Age |  |  |  |  |
| 15-29 yrs | 6,492 (38.1%) | 1,154 (50.5%) | 1,863 (42.2%) | 3,475 (33.6%) |
| 30-39 yrs | 6,286 (36.9%) | 661 (28.9%) | 1,536 (34.8%) | 4,089 (39.6%) |
| 40-49 yrs | 4,249 (25%) | 469 (20.5%) | 1,013 (23%) | 2,767 (26.8%) |
| Residence Type |  |  |  |  |
| Urban | 5,920 (34.8%) | 697 (30.5%) | 1,530 (34.7%) | 3,693 (35.7%) |
| Rural | 11,107 (65.2%) | 1,587 (69.5%) | 2,882 (65.3%) | 6,638 (64.3%) |
| Division |  |  |  |  |
| Barishal | 1,819 (10.7%) | 281 (12.3%) | 547 (12.4%) | 991 (9.6%) |
| Chattogram | 2,545 (14.9%) | 276 (12.1%) | 570 (12.9%) | 1,699 (16.4%) |
| Dhaka | 2,560 (15%) | 275 (12%) | 742 (16.8%) | 1,543 (14.9%) |
| Khulna | 2,229 (13.1%) | 390 (17.1%) | 569 (12.9%) | 1,270 (12.3%) |
| Mymensingh | 1,837 (10.8%) | 252 (11%) | 416 (9.4%) | 1,169 (11.3%) |
| Rajshahi | 2,172 (12.8%) | 237 (10.4%) | 582 (13.2%) | 1,353 (13.1%) |
| Rangpur | 2,082 (12.2%) | 218 (9.5%) | 492 (11.2%) | 1,372 (13.3%) |
| Sylhet | 1,783 (10.5%) | 355 (15.5%) | 494 (11.2%) | 934 (9%) |
| Highest Education |  |  |  |  |
| No education | 2,354 (13.8%) | 324 (14.2%) | 597 (13.5%) | 1,433 (13.9%) |
| Primary | 4,684 (27.5%) | 661 (28.9%) | 1,180 (26.7%) | 2,843 (27.5%) |
| Secondary | 7,677 (45.1%) | 1,067 (46.7%) | 2,038 (46.2%) | 4,572 (44.3%) |
| Higher | 2,312 (13.6%) | 232 (10.2%) | 597 (13.5%) | 1,483 (14.4%) |
| Number of Children |  |  |  |  |
| 0-1 child | 4,539 (26.7%) | 783 (34.3%) | 1,259 (28.5%) | 2,497 (24.2%) |
| 2 children | 6,707 (39.4%) | 797 (34.9%) | 1,762 (39.9%) | 4,148 (40.2%) |
| 3+ children | 5,781 (34%) | 704 (30.8%) | 1,391 (31.5%) | 3,686 (35.7%) |
| Wealth Index |  |  |  |  |
| Poorest | 3,089 (18.1%) | 459 (20.1%) | 832 (18.9%) | 1,798 (17.4%) |
| Poorer | 3,361 (19.7%) | 499 (21.8%) | 860 (19.5%) | 2,002 (19.4%) |
| Middle | 3,384 (19.9%) | 463 (20.3%) | 858 (19.4%) | 2,063 (20%) |
| Richer | 3,518 (20.7%) | 474 (20.8%) | 908 (20.6%) | 2,136 (20.7%) |
| Richest | 3,675 (21.6%) | 389 (17%) | 954 (21.6%) | 2,332 (22.6%) |
| Religion |  |  |  |  |
| Islam | 15,299 (89.4%) | 2,075 (90.8%) | 3,954 (89.6%) | 9,200 (89.1%) |
| Other | 1,798 (10.6%) | 209 (9.2%) | 458 (10.4%) | 1,131 (10.9%) |
| Respondent Working |  |  |  |  |
| No | 11,625 (68.3%) | 1,790 (78.4%) | 3,131 (71%) | 6,704 (64.9%) |
| Yes | 5,402 (31.7%) | 494 (21.6%) | 1,281 (29%) | 3,627 (35.1%) |
| Husband/Partner Working |  |  |  |  |
| No | 506 (3%) | 70 (3.1%) | 127 (2.9%) | 309 (3%) |
| Yes | 16,505 (97%) | 2,212 (96.9%) | 4,280 (97.1%) | 10,013 (97%) |
| BMI |  |  |  |  |
| Normal | 4,462 (52.5%) | 646 (56.5%) | 1,177 (53%) | 2,639 (51.5%) |
| Underweight or Overweight | 4,029 (47.5%) | 497 (43.5%) | 1,044 (47%) | 2,488 (48.5%) |
| Any Mental Health Symptoms |  |  |  |  |
| None | 11,609 (68.2%) | 1,631 (71.4%) | 2,894 (65.6%) | 7,084 (68.6%) |
| At least one symptom | 5,417 (31.8%) | 653 (28.6%) | 1,518 (34.4%) | 3,246 (31.4%) |
| Anxiety |  |  |  |  |
| No Anxiety | 13,758 (80.8%) | 1,901 (83.2%) | 3,578 (81.1%) | 8,279 (80.1%) |
| Anxiety | 3,269 (19.2%) | 383 (16.8%) | 834 (18.9%) | 2,052 (19.9%) |
| Depression |  |  |  |  |
| No Depression | 16,197 (95.1%) | 2,188 (95.8%) | 4,218 (95.6%) | 9,791 (94.8%) |
| Depression | 830 (4.9%) | 96 (4.2%) | 194 (4.4%) | 540 (5.2%) |

*^a^ Empowerment levels were calculated using the SWPER Global index and categorized using the original studies’ methodologies [12, 13]. Note: Some sociodemographic characteristics were not reported by every respondent; therefore, the total for these groups may not be equal to the total N = 17,027. Abbreviations: SWPER – Survey-based Women’s mpowerment; BMI – body mass index; GAD – Generalized Anxiety Disorder.*

**Table 2a.** Associations between women’s empowerment domains and depression: primary survey-weighted logistic regression and sensitivity analyses using survey-weighted Poisson regression, Bangladesh DHS

| Empowerment domain | Category | Adjusted OR (95% CI) | P value | Adjusted PR (95% CI) | P value |
| --- | --- | --- | --- | --- | --- |
| Attitude towards violence | High | 1.00 (Reference) | — | 1.00 (Reference) | — |
|  | Low | 1.81 (1.16–2.81) | 0.009 | 1.75 (1.19–2.58) | 0.004 |
|  | Medium | 1.14 (0.82–1.57) | 0.434 | 1.13 (0.84–1.52) | 0.419 |
| Social independence | High | 1.00 (Reference) | — | 1.00 (Reference) | — |
|  | Low | 0.75 (0.51–1.10) | 0.138 | 0.76 (0.53–1.08) | 0.126 |
|  | Medium | 0.77 (0.53–1.14) | 0.193 | 0.78 (0.55–1.12) | 0.181 |
| Decision-making | High | 1.00 (Reference) | — | 1.00 (Reference) | — |
|  | Low | 0.98 (0.70–1.37) | 0.896 | 0.98 (0.72–1.34) | 0.893 |
|  | Medium | 0.99 (0.75–1.30) | 0.944 | 0.99 (0.77–1.28) | 0.943 |

*OR, odds ratio; PR, prevalence ratio; CI, confidence interval.*

*All models were adjusted for age, place of residence, administrative division, educational attainment, number of children, household wealth quintile, religion, employment status, husband’s employment status, and body mass index. Models accounted for the BDHS sampling weights, stratification, and clustering using Stata survey commands. High empowerment was the reference category in every empowerment domain.*

**Table 2b.** Associations between women’s empowerment domains and anxiety: primary survey-weighted logistic regression and sensitivity analyses using survey-weighted Poisson regression, Bangladesh DHS

| Empowerment domain | Category | Adjusted OR (95% CI) | P value | Adjusted PR (95% CI) | P value |
| --- | --- | --- | --- | --- | --- |
| Attitude towards violence | High | 1.00 (Reference) | — | 1.00 (Reference) | — |
|  | Low | 1.46 (1.10–1.94) | 0.008 | 1.36 (1.12–1.65) | 0.002 |
|  | Medium | 1.26 (1.04–1.53) | 0.018 | 1.20 (1.05–1.38) | 0.009 |
| Social independence | High | 1.00 (Reference) | — | 1.00 (Reference) | — |
|  | Low | 0.88 (0.69–1.11) | 0.272 | 0.90 (0.75–1.08) | 0.253 |
|  | Medium | 0.87 (0.70–1.08) | 0.206 | 0.89 (0.75–1.06) | 0.193 |
| Decision-making | High | 1.00 (Reference) | — | 1.00 (Reference) | — |
|  | Low | 0.94 (0.77–1.16) | 0.580 | 0.95 (0.81–1.12) | 0.563 |
|  | Medium | 0.91 (0.78–1.06) | 0.213 | 0.92 (0.82–1.04) | 0.196 |

*OR, odds ratio; PR, prevalence ratio; CI, confidence interval.*

*All models were adjusted for age, place of residence, administrative division, educational attainment, number of children, household wealth quintile, religion, employment status, husband’s employment status, and body mass index. Models accounted for the BDHS sampling weights, clustering, and stratification using survey commands. High empowerment was the reference category for each empowerment domain.*

**Table 2c.** Associations between women’s empowerment domains and any mental health outcome: primary survey-weighted logistic regression and sensitivity analyses using survey-weighted Poisson regression, Bangladesh DHS

| Empowerment domain | Category | Adjusted OR (95% CI) | P value | Adjusted PR (95% CI) | P value |
| --- | --- | --- | --- | --- | --- |
| Attitude towards violence | High | 1.00 (Reference) | — | 1.00 (Reference) | — |
|  | Low | 1.63 (1.27–2.10) | <0.001 | 1.40 (1.22–1.60) | <0.001 |
|  | Medium | 1.56 (1.32–1.85) | <0.001 | 1.36 (1.23–1.49) | <0.001 |
| Social independence | High | 1.00 (Reference) | — | 1.00 (Reference) | — |
|  | Low | 0.87 (0.71–1.07) | 0.192 | 0.91 (0.80–1.04) | 0.176 |
|  | Medium | 0.88 (0.73–1.07) | 0.207 | 0.92 (0.81–1.04) | 0.195 |
| Decision-making | High | 1.00 (Reference) | — | 1.00 (Reference) | — |
|  | Low | 0.92 (0.77–1.10) | 0.356 | 0.94 (0.84–1.07) | 0.350 |
|  | Medium | 1.10 (0.97–1.26) | 0.136 | 1.07 (0.98–1.16) | 0.117 |

*OR, odds ratio; PR, prevalence ratio; CI, confidence interval.*

*All models were adjusted for age, place of residence, administrative division, educational attainment, number of children, household wealth quintile, religion, employment status, husband’s employment status, and body mass index. Models accounted for the BDHS sampling weights, stratification, and clustering using Stata survey commands. High empowerment was the reference category for each empowerment domain.*
